# Non-random mating within an Island rookery of Hawaiian hawksbill turtles: demographic discontinuity at a small coastline scale

**DOI:** 10.1098/rsos.221547

**Published:** 2023-05-17

**Authors:** John B. Horne, Amy Frey, Alexander R. Gaos, Summer Martin, Peter H. Dutton

**Affiliations:** ^1^ Southwest Fisheries Science Center, NOAA-Fisheries, La Jolla, CA, USA; ^2^ Pacific Islands Fisheries Science Center, NOAA-Fisheries, Honolulu, HI, USA

**Keywords:** conservation, sea turtles, breeding sex ratios, nesting frequency, inbreeding, Pacific Islands

## Abstract

Hawksbill sea turtles (*Eretmochelys imbricata*) from the Hawaiian archipelago form a small and genetically isolated population, consisting of only a few tens of individuals breeding annually. Most females nest on the island of Hawai'i, but little is known about the demographics of this rookery. This study used genetic relatedness, inferred from 135 microhaplotype markers, to determine breeding sex-ratios, estimate female nesting frequency and assess relationships between individuals nesting on different beaches. Samples were collected during the 2017 nesting season and final data included 13 nesting females and 1002 unhatched embryos, salvaged from 41 nests, of which 13 had no observed mother. Results show that most females used a single nesting beach laying 1–5 nests each. From female and offspring alleles, the paternal genotypes of 12 breeding males were reconstructed and many showed high relatedness to their mates. Pairwise relatedness of offspring revealed one instance of polygyny but otherwise suggested a 1 : 1 breeding-sex ratio. Relatedness analysis and spatial-autocorrelation of genotypes indicate that turtles from different nesting areas do not regularly interbreed, suggesting that strong natal homing tendencies in both sexes result in non-random mating across the study area. Complexes of nearby nesting beaches also showed unique patterns of inbreeding across loci, further indicating that Hawaiian hawksbill turtles have demographically discontinuous nesting populations separated by only tens of km.

## 1. Introduction

It is increasingly recognized that the maintenance of a population's size and corresponding genetic diversity is necessary for its long-term persistence [1–3]. Population decline often results in a significant loss of genetic diversity that leads to a cascade of detrimental effects such as inbreeding depression [2,4–7], loss of adaptive potential [8,9] and a reduced capacity for population recovery [10,–13]. For populations that remain small and isolated for prolonged periods of time the risk of extinction is high [3].

Most sea turtle populations worldwide have been dramatically reduced in size from historical levels, and virtually all still face challenges to persistence posed by a variety of human-related impacts [14–17]. The alarming rate of sea turtle decline has prompted several decades of conservation effort that has resulted in some cases of meaningful population recovery [18–21], suggesting that proper management can reverse negative trends and help ensure long-term persistence. However, not all populations have responded well to conservation protections and it is currently unclear what role genetic diversity may play in the ability of some sea turtle populations to rebound after experiencing demographic bottlenecks [22].

The hawksbill turtle (*Eretmochelys imbricata*) is a circumtropically distributed marine reptile with nesting colonies scattered across the Atlantic and Indo-Pacific. In the past, their numbers have dwindled for many of the same reasons as other sea turtle species, such as human harvesting for food, and fisheries bycatch, but also for being disproportionately targeted by the international shell trade, which is illegally ongoing in many countries [23]. Some hawksbill populations have shown signs of recent recovery [19,20] in response to management interventions, but others continue to decline in spite of efforts to protect their remaining numbers [24]. Currently, *E. imbricata* is listed as endangered under the US Endangered Species Act (ESA; [25]), and as critically endangered by the IUCN Red List [26].

In the Hawaiian archipelago, hawksbill turtles are described as rare and their resident population is thought to include less than 100 adult females, with only 5–26 nesting annually [27–29]. The population may also be predominantly female, as only 1 in 5 strandings in Hawaii are male [30]. Field surveys of nesting activity have been conducted since the 1980s with varying levels of effort, and predictive modelling based on these data suggests a recent uptick in the number of nesting females [29]. Nevertheless, the population remains extremely small, despite having endangered species status in the USA since 1970 (https://www.federalregister.gov/citation/35-FR-8491).

Given the vulner­ability of Hawaiian hawksbill turtles, due to their chronically low abundance, and the fact that they harbour unique mitochondrial genetic diversity for this species [31], there is motivation to better understand the conservation needs of this population. Monitoring these turtles is difficult, however, because outside of the breeding season they disperse across hundreds of km to various foraging areas throughout the archipelago, often crossing deep ocean channels between islands [30,32]. During the breeding season is the easiest time to observe females, but nesting takes place at multiple remote and hard-to-access beaches, most of which are located along the southern coast of Hawai'i Island [29]. Females lay up to 250 eggs that hatch after 50–65 days, but the nesting habits of individuals are poorly understood, as many nests are only discovered after the mother has returned to the ocean and field efforts have been unable to confidently census nesters. A consequence of these uncertainties is that the mating system of Hawaiian hawksbills, and their operational breeding sex ratios are unknown.

One way to fill demographic information gaps for Hawaiian hawksbill turtles is with genetic relatedness analysis. Using genetic markers, it is possible to infer parent-offspring relationships between known nesting females and nests where the mother is unidentified [33,34] and obtain a more complete population census. The genetic relationships of offspring within and among nests also indicate patterns of paternity, and can be used to obtain information on mating systems and breeding sex ratios [35–37]. In this study, an amplicon-based array of single nucleotide polymorphisms (SNPs), and small nucleotide insertion–deletion (indel) markers, was developed to capture population-specific variation in Hawaiian hawksbill turtles and to estimate pairwise genetic relatedness. The data presented hereafter describe the organization of genetic diversity within the Hawaii Island rookery, provide insight into the basic reproductive biology of a precariously endangered population of sea turtles, and also show that such studies can yield unexpected results with importance to conservation management.

## 2. Methods

### 2.1. Sample collection and study design

Tissue samples from nesting females were collected during the 2017 breeding season at five nesting beaches located along the southern coast of Hawai'i Island. Samples consisted of a skin biopsy (approx. 0.5 cm^2^) taken from the neck or shoulder after nesting. Nests were marked immediately after being laid, or after nesting turtle tracks were encountered during beach patrols (unobserved females). Tissue was stored in a high-salt solution for transport. Offspring tissue collections were flippers of dead embryos salvaged from 41 nests (table 1) after live hatchlings had vacated the nesting chamber. For 13 of these nests, the mother was not observed during nesting and is unknown. Ten females from the 2018 nesting season were included in this study to act as control samples for assigning mothers to nests. Female Hawaiian hawksbills are not known to nest in consecutive years [29], so it is presumed that these off-year individuals could not have been the unidentified mothers and thus serve as negative controls. Inconel flipper tags (National Band & Tag, Newport, KY, USA) and Passive Integrated Transponder (PIT; Avid, Norco, CA, USA) tags are applied to all female turtles encountered to confirm and track identity during nesting seasons. One turtle, nesting mother-160, did not have an available DNA sample for genotyping.

**Table 1.** Hawai'i Island hawksbill turtle nests from 2017, with the nesting complex (figure 3*a*), number of offspring genotyped per nest, observed mother, inferred mother from parentage analysis and probability of maternity. Inferred mothers marked with an asterisk were not fully supported by all analyses. Note: no genetics sample of mother-160 was available for this study.

nest	nesting complex	offspring genotyped	observed mother	inferred mother	prob.
Apua-01	‘Āpua	19	153	153	1.000
Apua-02	‘Āpua	46	153	153	1.000
Apua-03	‘Āpua	4	155	155	1.000
Apua-06	‘Āpua	25	153	153	1.000
Apua-07	‘Āpua	21	none	153	1.000
Apua-08	‘Āpua	62	158	158	1.000
Apua-09	‘Āpua	51	154	154	1.000
Apua-10	‘Āpua	4	155	155	1.000
Apua-11	‘Āpua	16	153	153	1.000
Apua-12	‘Āpua	16	none	158	1.000
Apua-14	‘Āpua	37	155	155	1.000
Apua-15	‘Āpua	95	none	158	1.000
Apua-16	‘Āpua	21	none	158	1.000
Halape-01	‘Āpua	72	none	85	1.000
Halape-02	‘Āpua	10	85	85	1.000
Halape-03	‘Āpua	76	85	85	1.000
Pohue-01	Pōhue	4	none	119	0.991
Pohue-02	Pōhue	1	151	151	1.000
Pohue-03	Pōhue	19	152	152	1.000
Pohue-04	Pōhue	2	119	119	0.991
Pohue-05	Pōhue	9	71	71	1.000
Pohue-06	Pōhue	30	76	76	1.000
Pohue-07	Pōhue	8	151	151	1.000
Pohue-08	Pōhue	6	152	152	1.000
Pohue-09	Pōhue	8	none	157	1.000
Pohue-10	Pōhue	14	119	119	0.991
Pohue-11	Pōhue	48	76	76	1.000
Pohue-12	Pōhue	15	71	71	1.000
Pohue-13	Pōhue	1	151	151	1.000
Pohue-15	Pōhue	18	152	152	1.000
Pohue-17	Pōhue	17	71	71	1.000
Pohue-18	Pōhue	8	151	151	1.000
Pohue-19	Pōhue	20	76	76	1.000
Pohue-20	Pōhue	23	none	76	1.000
Pohue-21	Pōhue	7	152	152	1.000
Pohue-24	Pōhue	8	none	159*	0.687
Pohue-25	Pōhue	3	160	159*	0.687
Awili-01	Pōhue	54	none	157	1.000
Awili-02	Pōhue	59	none	157	1.000
Koloa-01	Kamehame	10	none	110	1.000
Koloa-02	Kamehame	23	none	110	1.000

### 2.2. Marker development

Because the power of genetic parentage analysis improves more with increased marker polymorphism and heterozygosity than the total number of markers [38], this study sought to develop a panel of 100–300 polyallelic microhaplotype loci (see [39–41]) to capture genetic variation specific to Hawaiian hawksbill turtles. Candidate loci for a PCR-amplicon array were chosen from double-digested restriction site associated DNA sequences (ddRADseq; [42]) from 18 Hawaiian hawksbills. Individual DNA concentrations were normalized to 500 ng, and samples were digested with two independent sets of two enzymes: set one (library 1) included EcoR1 and Sph1, and set two (library 2) included MluC1 and Sph1. Genomic fragments between 400 and 600 bps were excised from a 2% Agarose gel. All other details of the library preparations were performed as in Peterson *et al.* [42]. Libraries were sequenced on an Illumina MiSeq sequencing platform using a paired-end approach and a 600 cycle sequencing kit. The pipeline STACKS v. 1.34 [43] was used to demultiplex raw reads and identify allelic variants, requiring a minimum stack depth of 4 , a distance of 4(M) allowed between stacks and a distance of 4(n) allowed between catalogue loci. A total of 63 610 unique genomic segments were found, of which 2439 held at least two SNPs for at least five individuals. Loci were also screened for the density of polymorphisms at 100–300 bp intervals. Three hundred and seventeen genomic segments were randomly selected from the remaining Stacks loci for amplicon design, and PCR primers were designed for 259 of the candidate loci using FastPCR software [44]. Following four rounds of panel optimization, and the removal of paralogous sequences, 229 loci remained in the final panel.

### 2.3. DNA extraction, PCR amplification and DNA sequencing

Genomic DNA was extracted from tissue using a sodium chloride extraction (modified from [45]). Extracted DNA concentrations were normalized to 10 ng ul^−1^. A total of 1242 individuals were included for PCR amplification and sequencing, plus 180 replicates to assess genotyping consistency, and one negative control with no template DNA for every 96-well plate.

The genotyping-in-thousands by sequencing protocol (GT-seq) of Campbell *et al*. [46] was used to generate sequence data for genotype calling. An initial multiplex PCR containing locus-specific primers with Illumina (Illumina, Inc., San Diego, CA, USA) priming sites for 229 amplicons was performed, followed by a second PCR to add Illumina adapters with indexes. Amplicon DNA concentrations were normalized across samples following PCR 2, using SequalPrep Normalization Plate Kits (Thermo Fisher Scientific), and pooled for a purification step performed using Agencourt AMPure XP magnetic beads to size select PCR products for sequencing. Each purified pool was quantified by Qubit Fluorometer (Thermo Fisher Scientific), and then by qPCR with the Illumina Library Quantification Kit (Kapa Biosystems). Pools were normalized to 4 nM and pooled together. The library was sequenced on an Illumina Nextseq 500 using a single-end approach and a 150-cycle sequencing kit. All other details of the thermal cycling and library preparation are as in Campbell *et al*. [46].

### 2.4. Data processing, SNP genotyping and microhaplotying

Adapter sequences, and base-pairs with a Phred quality score less than 15, were trimmed from fastq reads using the program FASTP v. 0.23.1 [47]. Sequences smaller than 90 bp after trimming were also excluded. Trimmed reads were then aligned to a fasta reference using the program BOWTIE2 v. 2.3.4.1 [48]. Alignments were then sorted and indexed using SAMBAMBA v. 0.7.1 [49] while requiring a mapping quality score of greater than or equal to 20. Sample alignments with fewer than 10 000 mapped reads were determined to have suboptimal amplicon sequencing depth and were excluded from further processing.

To reduce software biases introduced during genotyping (see [50–53]) and improve genotyping accuracy [54], variant calling was performed independently using three programs: BCFTOOLS v. 1.9 [55], FREEBAYES v. 1.3 [56] and GATK-HC v. 3.8 [57]. Sensitivity was maximized for all three callers by accepting low alternate allele fractions (FREEBAYES), applying read fractions to individual rather than pooled samples (BCFTOOLS), and disabling pruning algorithms (GATK-HC). Called variants were reduced to their simplest components using the vcfallelicprimitives script from VCFLIB [58]. To minimize the trade-off between genotyping sensitivity and accuracy, variants with low genotyping fidelity were identified using sequencing replicates and discarded if they had mismatched allele calls in greater than 7% of replicate sample pairs. In addition, variants were discarded if they were not identified by at least 2 out of the 3 variant callers, or had greater than 5.5% allelic mismatches among callers. The 7 and 5.5% mismatch thresholds were chosen after viewing histograms of allelic mismatches and making a qualitative determination about the level of genotyping noise (from unavoidable PCR and sequencing errors) that should be tolerated as normal (electronic supplementary material, figure S1). The custom R functions that performed the mismatch comparisons are available online (github.com/jh041/loc_gen_acc). SNPs and small indels that were robust to genotyping errors were then filtered for minor allele frequency and missing data using VCFTOOLS v. 0.1.16 [59]. Any variants with a minor allele frequency less than 0.01 were removed. Another experimental version of the data was also produced that required a minor allele frequency threshold of 0.05 and only allowed binary SNPs. Filtering for missing data followed an iterative approach, gradually decreasing missing data allowances from 80% to 30% for both loci and individuals. Variants from the three callers were filtered separately and not combined into a single dataset until after microhaplotyping.

Microhaplotyping was performed using the R package MICROHAPLOT v. 1.0.1 (https://github.com/ngthomas/microhaplot) that uses both genotype calls and mapped reads to produce short phased haplotypes of all genetic variation found on each PCR amplicon. Additional filtering parameters were applied to microhaplotypes using MICROHAPLOT's R Shiny app., including a minimum total microhaplotype read depth of 12, and an initial minimum allelic ratio of 0.50 (the minor microhaplotype allele must have a depth at least one half that of the major allele). Afterwards, the allelic ratios were refined for each amplicon locus individually by examining the relative depths of alleles for homo and heterozygous microhaplotype calls. The acceptable allele ratio for a homozygous call was never more than 0.09. The acceptable allele ratio for a heterozygous call was never less than 0.20. A minimum fraction of 0.7 microhaplotypes with acceptable allelic ratios across all individuals was required for each amplicon. If any individual had more than two possible microhaplotypes for the same locus (possibly indicating DNA contamination) the locus or sample was either removed, or noisy low-frequency microhaplotypes were excluded. At this point, microhaplotype calls arising from the FREEBAYES, BCFTOOLS and GATK_HC outputs were combined into a single dataset. Microhaplotype loci were removed from the analysis if more than 7% of allele calls were mismatched with replicate samples. Linkage among amplicon microhaplotypes (LD) and departures from Hardy–Weinberg expectations (HWE) were assessed using the program GENEPOP [60] and GENODIVE v. 3.05 [61] adjusting *p*-values for multiple hypothesis testing using the method of Benjamini & Hochburg [62].

### 2.5. Genetic diversity and relatedness analysis

Genetic diversity statistics for our samples, including heterozygosity and *G*-statistics, were calculated for the final dataset using GENODIVE. Pairwise relatedness coefficients (*r*) were computed for all turtles with the R package RELATED v. 1.0 [63] using sample allele frequencies as reference points for the calculation of allelic states. The analysis is sensitive to genetic stratifications and linkage disequilibrium [64], therefore, if two loci were shown to be in linkage disequilibrium then one of them was removed before relatedness analysis, and a number of different sample subsets were experimentally used to explore the sensitivity of the analysis to changes in the sample reference. The relative performances of all relatedness estimators available from RELATED were evaluated using the native simulation modules for this package, generating 100 simulated genotypes each of four relatedness classifications (parent-offspring, full-sibling, half-sibling and unrelated). The estimator with the best correlation between simulated and inferred coefficients across all relatedness classes was then used to compute *r* for the empirical data. Final analysis was run with 1000 bootstrap replicates to generate 95% confidence intervals for each pairwise value, and used an error-rate parameter of 0.02 for each locus. Inbreeding was set to ‘allowed'.

Pairwise relatedness was also inferred using the R package CKMRsim v. 0.1 [65], which uses a pseudo-likelihood approach and forward-in-time Monte Carlo simulations to reconstruct pedigrees based on data-specific allele frequencies, from which different types of relatedness can be modelled. In theory, this approach is less sensitive than relatedness coefficients to structures in the sample allele frequencies, because these become incorporated into the model, such that even distant relationships can be referenced against an underlying pedigree, but power is still compromised by linkage disequilibrium between loci [41]. The results of this analysis were validated with 10 000 simulated genotype pairs of each relatedness type, generated using CKMRsim's model framework. Simulated data were used to determine expected type-I and type-II error rates and baseline ranges of log-likelihood ratios for the following relatedness tests: parent-offspring versus unrelated, full-sibling versus unrelated, half-sibling versus unrelated and full-sibling versus half-sibling. This analysis was also run with an assumed 2% error rate per locus.

Lastly, relatedness analysis was performed in COLONY v. 2.0.6.6 [66], which differs from the other relatedness analyses by inferring the full pedigree likelihood of all samples simultaneously, instead of relying on pairwise inferences of relatedness. This analysis also uses pedigree information to assess genotyping error rates for each locus and reconstruct the pedigrees of unknown parents, such as the unsampled male hawksbill sires in this study. The paternal genotypes imputed from COLONY2 analysis were incorporated into all previously mentioned analyses as additional samples. Inbreeding and polygamy was allowed in the analysis. COLONY2 also estimates the effective population size (*N*_e_) of the breeding population using the sibship assignment method [67].

Due to small population size, a strong signal of background inbreeding was suspected for Hawaiian hawksbill turtles, which can confound the accuracy of standard pairwise relatedness metrics [68,69]. Though some of the used relatedness calculations have methods to reduce inbreeding biases implicit in the data (e.g. COLONY2), whether these were sufficient for the target population was not known *a priori*. However, because there were known relationships in our data (i.e. observed nesting females and offspring, full-sibling nest-mates) we relied on relatedness inferences between these individuals to determine if inbreeding was adversely impacting relatedness estimates.

### 2.6. Spatial patterns of genetic variation

In addition to relatedness inferences, several methods available in the R package ADEGENET [70] were used to assess spatial allelic patterns using multivariate statistics that do not assume loci are in linkage equilibrium, or in Hardy–Weinberg proportions, and which are not sensitive to signals of selection or inbreeding. First, the data from 2017 nesting females and reconstructed paternal genotypes were clustered according to a *K*-means clustering algorithm, using 20 principal components as predictors. Discriminant analysis of principal components (DAPC: [71]) was then used to give a multivariate ordination of genetic differentiation based on these clusters. Finally, whether there was any positive spatial autocorrelation of genotypes among nesting sites was assessed using a spatial principal components analysis [72]. All individuals were georeferenced with latitude and longitude coordinates corresponding to nesting beaches where their offspring were born, with a slight amount of jitter added to avoid replicate coordinates. Statistical support for the result was determined using a Monte Carlo procedure (the global *r*test included in the ADEGENET package) and 1000 permutations. The *p*-value of this test indicates the proportion of permuted statistics that exceed or are equal to the maximum observed value.

## 3. Results

### 3.1. Data processing, and genetic diversity

The mean number of raw, unmapped fastq DNA sequence reads per turtle was 366 235. Out of 1422 raw fastq files, 257 were discarded for having less than 10 000 mappable reads, leaving 1165 for analysis. The mean number of mapped reads across 1165 samples was 271 490. The mean sequencing depth of PCR amplicons in the multiplex ranged from 22 to 7500, with most having depths between 300 and 500. Of the 1165 mapped samples, 140 were replicates used to assess the consistency of genotype calls. The final data included 1002 offspring from 41 nests, 13 adult females from the 2017 breeding season (tables 1 and 2) and 10 control females from the 2018 breeding season.

**Table 2.** Nesting female hawksbill turtles from the 2017 nesting season, with the nesting beaches used, number of nests, total number of unhatched offspring collected and mean per nest, as well as the genotype ID for the inferred males constructed using offspring genotypes, and the estimated relatedness coefficient between the mating pair [73]. Note: no genetics sample of mother-160 was available for this study.

nesting female	nesting beach	number of nests	unhatched offspring	mean unhatched offspring per nest	mates	mate pairwise relatedness
153	‘Āpua	5	162	32.4	P01	0.25
154	‘Āpua	1	55	55	P04	0.15
155	‘Āpua	3	58	19.3	P02	0.14
158	‘Āpua	4	238	59.5	P03	0.24
85	Halapē	3	168	56	P06	0.28
119	Pōhue	3	31	10.3	P07	0.27
151	Pōhue	4	33	8.25	P09	−0.06
152	Pōhue	4	83	20.7	P10	−0.05
157	‘Āwili	2	127	42.3	P05	0.09
	Pōhue	1				
71	Pōhue	3	49	16.3	P11	0.0
76	Pōhue	4	138	34.5	P07	0.07
110	Kōloa	2	44	22	P8	0.27
160/159	Pohue	2	30	10	P12	−0.04

The three variant calling software programs each returned different numbers of raw SNP and indel DNA polymorphisms (BCFTOOLS = 1839, FREEBAYES = 971 and GATK = 766). A total of 2201 variants were detected by all programs. After removing variants with poor genotyping consistency across replicate samples and between variant calling programs, and filtering for missing data, there were 281, 202 and 256 variants from each of the callers, respectively. Only one of the final variants was an indel. Binary SNPs made up 85–98% of the other variants with the rest being trinary or quaternary SNPs. The final mean numbers of variants per PCR amplicon were 1.66, 1.72 and 1.58, for each of the callers, respectively (electronic supplementary material, table S1). Preliminary analyses using only binary SNPs and a minor allele frequency threshold of 0.05 returned results consistent with the final dataset that included all variant types.

**Figure 1.**
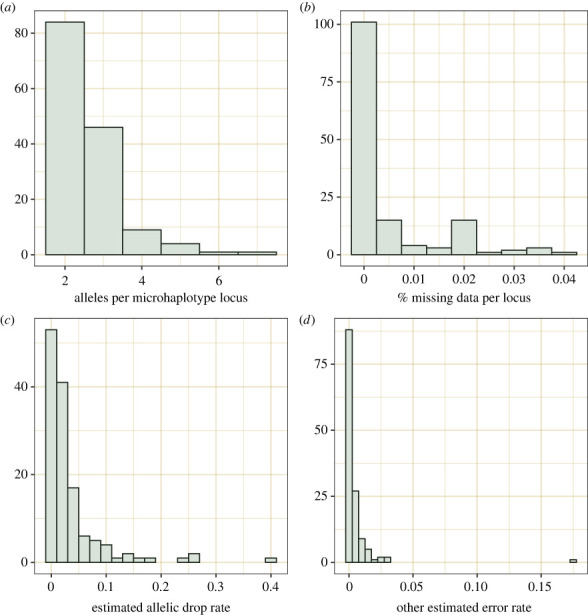
Descriptive statistics for 135 microhaplotye loci from 1026 hawksbill turtles. (*a*) Alleles per locus, (*b*) the proportion of missing data per locus, (*c*) estimated allelic dropout rate per locus and (*d*) estimated rate of other errors per locus.

A total of 170 PCR amplicons from all three callers were used for microhaplotyping and 135 final microhaplotype loci passed all filtering parameters, including linkage disequilibrium. The mean number of microhaplotype alleles per locus was 2.61, and the maximum number of alleles was seven (figure 1*a*). The mean missing data across all markers was 0.5% (figure 1*b*). The mean genotyping consistency of the final marker panel across replicate samples was 97.7%, and only two microhaplotype loci needed to be removed for having less than 93% consistency. Genotyping error rates estimated by COLONY2 suggest that the mean allelic drop rate across all markers was 3.5% (95% CI = 1.77%–6%), and the mean rate of all other errors was 0.4% (figure 1*c,d*). Estimated allelic drop rates were associated with the amount of missing data in each locus.

**Figure 2.**
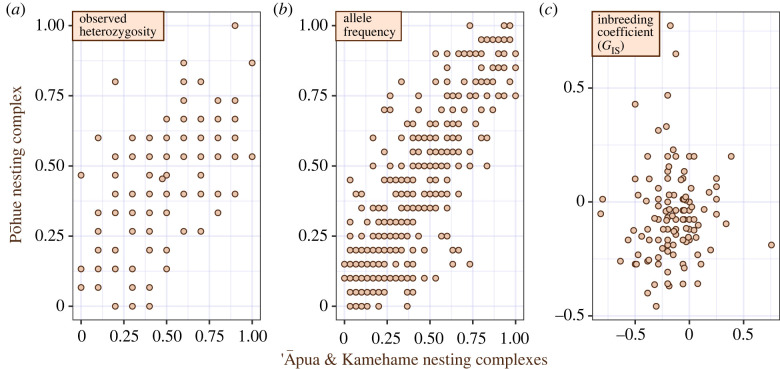
Per-locus population genetic comparisons of the Pohue nesting complex versus the ‘Āpua and Kamehame nesting complexes: (*a*) observed heterozygosity, (*b*) allele frequency and (*c*) the inbreeding coefficient *G*_IS_ of nesting females and inferred paternal male genotypes.

**Figure 3.**
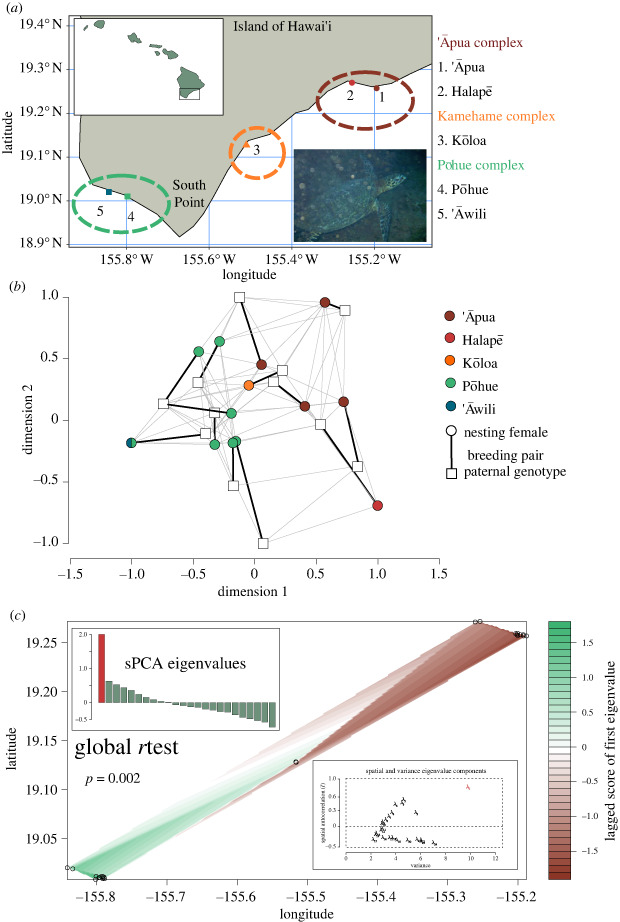
(*a*) Map of the study area and the three nesting complexes. Photo credit: John B. Horne (*b*) relatedness network. Nodes represent nesting mothers and the inferred genotypes of their mates, reconstructed from hatchling microhaplotype loci. Edges are multi-dimensionally scaled pairwise relatedness values (1 − *r*). Edges smaller than *r* = 0 are omitted. (*c*) Interpolated map of individual scores from spatial principal components analysis. Inserts depict the relative contributions of each eigenvalue to the spatial autocorrelation of genetic variation.

Patterns of genetic diversity differed between nesting complexes, especially across the southern point of the island (South Point) separating the ‘Āpua and Kamehame complexes in the east from the Pōhue complex in the west (figures 2 and 3), with some loci having fixed alleles across this divide.

The per-locus heterozygosity estimates for east and west nesting complexes were similar, and show a positive, though loose, correlation when plotted against each other (figure 2*a*). A similar relationship was seen for allele frequencies (figure 2*b*). The variance around these positive associations represents genetic differentiation that in terms of population structure was estimated at $G_{\mathrm{ST}}^{\prime }=0.036$, *p* < 0.005; $G_{\mathrm{ST}}^{\prime \prime }=0.061$, *p* < 0.005; and Jost's *D* = 0.027, *p* < 0.005. When only the 12 most polymorphic microhaplotype loci, with four or more alleles, were assessed for population structure the indices were: $G_{\mathrm{ST}}^{\prime }=0.062$, $G_{\mathrm{ST}}^{\prime \prime }=0.152$, Jost's *D* = 0.06, with all values being statistically supported at *p* < 0.005.

In contrast to other metrics of genetic diversity, and fixation indices, the locus-specific inbreeding coefficients (*G*_IS_) for both the east and west nesting complexes had no discernible relationship when plotted against each other (figure 2*c*). Values for both complexes ranged between −0.7 and 0.7, with mean values falling below zero in both cases, and being significantly different from zero after 1000 bootstrap replicates. In spite of many extreme *G*_IS_ values, departures from HWE were not statistically supported for any locus when both nesting complexes were analysed jointly.

### 3.2. Relatedness analysis

**Figure 4.**
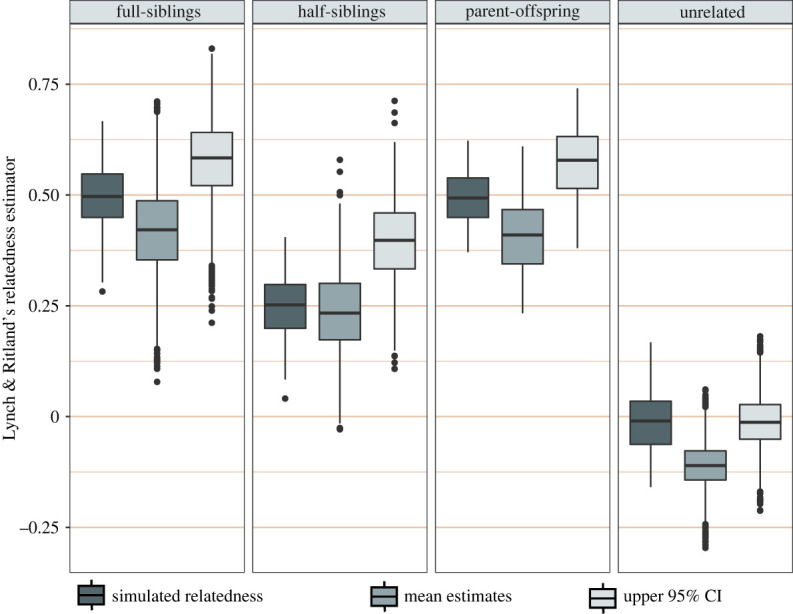
Boxplots showing the ranges of pairwise relatedness values [73] for full-siblings, half-siblings, parent-offspring pairs and unrelated individuals. Simulated ranges were calculated from 100 simulated pairs for each relationship type. Observed ranges of parent-offspring and full-sibling relatedness are from individuals with known relationships. Mean and 95% confidence intervals were produced after 1000 bootstrap replicates.

The best method-of-moments relatedness estimator for our data was that of Lynch & Ritland [73], being over 94% correlated with the simulated levels of relatedness, and is hereafter referred to as *r*. The *r* distributions of known pairwise parent-offspring and full-sibling relationships from this study overlapped with simulated ranges but empirical *r* values tended to be lower than simulated data (figure 4). Changing the reference allele frequencies (by including or excluding offspring, 2018 nesters, or only using allele frequencies from the same nesting complex) did little to alleviate the skew. Nevertheless, whenever mean *r* was low the upper 95% confidence limit was helpful as a secondary measure for comparing pairwise relatedness to simulation expectations (figure 4).

**Figure 5.**
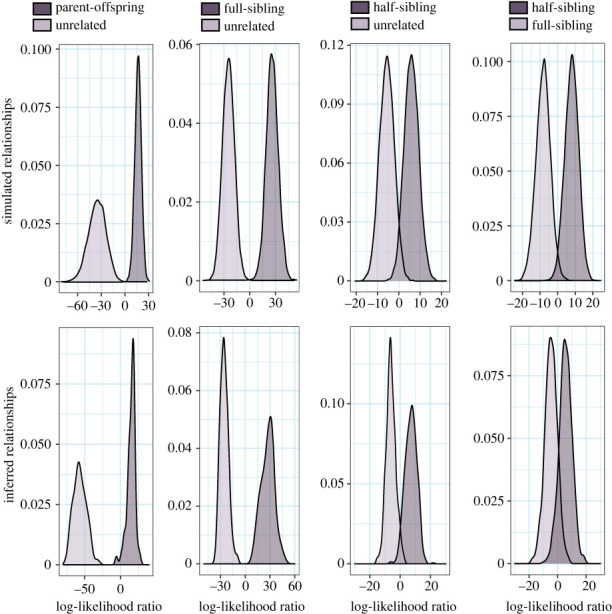
Kernel density plots of log-likelihood ratios for four different relationship comparisons. Top row: the expected log-likelihood ratio distributions for parent-offspring, full-sibling and half-sibling pairs compared to unrelated individuals, from 10 000 simulated individuals for each category. Bottom row: observed log-likelihood ratios for each category. Distributions of parent-offspring and full-sibling ratios are from individuals with known relationships.

Log-likelihood ratios for known parent-offspring and full-sibling relationships were approximated by simulated distributions (figure 5). In general, methods-of-moments and likelihood-based relatedness methods were in strong agreement with each other, and both were concordant with COLONY2 results. Every nesting female with a known nest was correctly identified by all three analyses as the mother of the offspring (table 1). PIT logs also confirmed that each inferred nesting mother was in the general area when the nest was laid. Likewise, a full-sibling relationship was confirmed for every nest-mate pairing, as well as between offspring from different nests laid by the same mother. These clear and predictable results affirm the effectiveness of our methods and suggest that potentially confounding factors such as generational inbreeding, and negative selection bias (from targeting dead offspring exclusively), have not adversely affected the outcome of these analyses.

Offspring from nests with an unknown mother had clear parentage with a single candidate female in all but two cases (table 1). The only exceptions were nests Pohue-24 and -25, where COLONY2 indicated mother-159 as the best mother for both with only 68.7% confidence, and the other methods were equally inconclusive. However, nest Pohue-25 is known to have been laid by mother-160, which was not sampled for this study but may be closely related to mother-159. Given that the same female most likely laid nests Pohue-24 and -25, the data show that 13 nesting females were responsible for the 41 sampled nests. Nesting females laid 1–5 nests with the mean being 2.9 (table 2). Mother-157 was the only female to use multiple nesting sites within the same nesting complex, with two nests at ‘Āwili beach and one at Pōhue beach.

Paternal genotype reconstructions in COLONY2 indicate that there were 12 breeding males for 13 breeding females, with father P07 having mated with mother-119 and mother-176 (table 2 and figure 3*b*). Examination of pairwise relatedness between the offspring of these two nesting females corroborates this, indicating that they are half-siblings. Therefore, the operational breeding sex ratio for Hawaiian hawksbill turtles in 2017 appears to be near parity, with some female bias in the Pōhue nesting complex. There was no evidence of any half-sibling relationships among any of the nest-mates, and no indication of polyandry during the 2017 breeding season. Mean estimates of N_e_ from COLONY2 were 8 and 16 for non-random and random mating scenarios, respectively. The upper and lower 95% confidence bounds for N_e_ ranged from 4 to 34 across all estimates.

One unexpected result from the pairwise relatedness coefficients was that mean *r* for all the nesting females was noticeably less than the mean *r* values for the east and west nesting complexes individually. Closer inspection revealed that turtles breeding on opposite sides of South Point, both males and females, tended to be more related to each other than to turtles nesting across the point (figure 3*b*). Many breeding pairs also had high pairwise *r* values between them, especially from the ‘Āpua and Kamehame nesting complexes (table 2). These data indicate that the fidelity of Hawaiian hawksbills to specific natal breeding areas has resulted in a spatial pattern of non-random mating that is functionally equivalent to assortative mating, and that inbreeding is not avoided when unrelated mates are accessible at other nearby breeding areas.

### 3.3. Multivariate population genetic analyses

The K-means clustering algorithm implemented in ADEGENET split nesting females and inferred paternal genotypes between eastern and western nesting complexes, corroborating genetic diversity indices and relatedness analysis, and suggesting that these genetic differences are biologically meaningful. Genetic differences may also separate the eastern ‘Āpua and Kamehame nesting complexes, the latter of which was poorly sampled in the current study, but the available data indicate that the strongest genetic differentiation divides Hawai'i Island nesting turtles across South Point (figure 3). In both sPCA and DAPC analysis, a major proportion of the conserved genetic variance was spatially segregated across South Point (figure 3*c*). Thus, while both male and female hawksbill turtles are observed throughout the Hawaiian archipelago [30], to reproduce they appear to sort geographically according to nesting complex.

## 4. Discussion

Though all sea turtles exhibit natal homing behaviours, there is considerable variation among species, populations, and individuals in nesting site fidelity. Female leatherback turtles (*Dermochelys coriacea*), for example, are not as strictly philopatric as other species [74], and individuals display a range of nesting behaviours with some laying nests over 400 km apart in a single nesting season [75]. Hawksbill females, by contrast, are among the more faithful of sea turtles to their natal nesting beaches [76–79], but many populations are genetically connected through male-mediated gene flow, particularly when rookeries are located along the same coastline [80,81]. Compared to what has been reported for hawksbills in other parts of the world, the level of genetic differentiation observed among Hawaiian nesting complexes was high and unexpected.

Not only was there significant genetic population structure detected within the study area, but relatedness analysis revealed that both males and females appear to be mating assortatively by nesting complex (figure 3). The coastline distance between complexes (less than100 km) is well within the dispersal capabilities of adult Hawaiian hawksbills, which are known to migrate for hundreds of km and cross the deep ocean channels between islands [32]. Therefore, these turtles appear to breed with other members of their same nesting complex even when other mating opportunities are available. Population structure ($G_{\mathrm{ST}}^{\prime }$) is likely exacerbated by small population size, and the positive associations between per-locus heterozygosity and allele frequency from the different complexes suggests some degree of long-term genetic connectivity or shared ancestry (figure 2*a,b*). However, contrasting signals of inbreeding and outbreeding in different nesting complexes at each locus (figure 2*c*) indicate that non-random mating is not unique to the 2017 nesting season alone, because this pattern would require generations to form. Additionally, mean *G*_IS_ values would return to zero after only one generation of random mating among groups. More data will be needed to make sense of the distinct east-west inbreeding patterns, but one explanation could be that Hawaiian hawksbill nesting colonies are currently trying to balance trade-offs between inbreeding and outbreeding depression [82].

Inbreeding and outbreeding depression require an association between genetic diversity and reproductive fitness. In sea turtles, one measure of this fitness is likely the successful hatch rate of eggs. In the present study, all sampled offspring were unsuccessful hatches, and the rate of successful hatches was assessed from empty egg shells in the nest chamber (electronic supplementary material, table S2). These data from the 13 females included in this study were not large enough for robust statistical analysis, but a study by Phillips *et al*. [83] that was able to sample 95 nesting hawksbill females from the Seychelles, and their offspring, found evidence of both positive and negative correlations between multi-locus heterozygosity and hatching success, which suggests tension between inbreeding and outbreeding depression. Future studies of Hawaiian hawksbills, and other threatened populations of marine turtles, should pay closer attention to the relationship between genetic diversity and reproductive success, this being an aspect of their biology that is poorly understood but consequential for favourable conservation outcomes [22].

For breeding units as small as the nesting complexes of Hawaiian hawksbill turtles, even infrequent gene flow between them may be enough to prevent the loss of genetic diversity and stave off the worst effects of inbreeding [3,84–86]. This is because in a subdivided population random genetic drift will cause alleles to equilibrate differently in the different subunits, and the smallest subunits will experience the strongest genetic drift [87]. Gene flow between population subunits with different drift loads can yield substantial heterosis benefits, which are maximized when population sizes are small and migration between them is low [84]. A loosely connected metapopulation of small cohesive breeding units would also theoretically be able to purge deleterious alleles more effectively, and potentially be more stable than if all nesting complexes formed a single randomly mating population unit [86]. Testing such a hypothesis for sea turtles would be difficult, but this could help explain why some populations appear perpetually small but steady over extended periods of time.

The mating system of Hawaiian hawksbills may also be important for genetic load management in this small population. This is because assortative mating within breeding groups can allow lineages to purge deleterious mutations more efficiently, in a similar fashion as subdivided populations [88]. In other parts of the world, hawksbill breeding sex ratios can be heavily female biased, presumably due to a limited supply of males in small populations [89], but the Hawaiian rookeries have even fewer breeding individuals than elsewhere and a nearly 1 : 1 sex ratio, notwithstanding that females may outnumber males 4 : 1 overall in the archipelago [30]. Therefore, it cannot be ruled out that Hawaiian hawksbills are highly selective in their mate choices, even within nesting complexes (figure 3*b* and table 2; see next section).

Because of the demographic discontinuity between nesting complexes there could in reality be multiple mating systems, and differences in breeding sex ratios, within the Hawaiian hawksbill metapopulation. At the outset of this research this degree of population complexity was not anticipated and more samples will be needed to elaborate on the patterns uncovered in this work. For example, more turtles from the Kamehame complex are required to determine genetic structure with nearby ‘Āpua (figure 3), and there are other nesting areas in the main Hawaiian Islands that could be genetically and demographically unique. Most nesting sites not on the island of Hawai'i are extremely low-density, having less than two nests laid annually, but one rookery on the island of Molokai has at least as many nesting females as any of the Hawai'i Island nesting complexes [29]. Samples from multiple breeding seasons are also needed to better understand mating systems and female nesting behaviour within and between complexes.

### 4.1. Skew in estimates of pairwise relatedness

The genetic drift and mating system that determine the level of structure between two populations also determine the level of relatedness between two individuals, thus genetic population structure and genetic relatedness are two different aspects of the same variation [90]. Just as measures of population structure can be seen as coancestry averages between the individuals in populations [91], relatedness is a measure of coancestry between two individuals [92]. The inextricable connection between genetic structure and genetic relatedness means that patterns in one are relevant to patterns in the other.

An overabundance of negative relatedness coefficients was observed for unrelated Hawaiian hawksbill turtles, and the *r* values of many related individuals were also depressed compared to distributions simulated under optimal conditions (figure 4). For the purposes of distinguishing related turtles from unrelated ones this skew is just noise, because all relatedness methods were able to correctly identify known relationships, regardless. But given the degree of inbreeding revealed by this study the skew deserves further exploration because negative pairwise *r* values are expected for samples that are outbred (have fewer loci that are identical-by-descent) relative to the reference allele frequencies [93,94].

One source of skew in the data could be genetic structure in the reference allele frequencies, which can negatively bias relatedness estimates [64,94]. Significant genetic structure was found between nesting complexes, but the skew in *r* values persisted even when the reference was generated exclusively from the same nesting complex. For the eastern nesting complexes, structure between Kamehame and ‘Āpua could be partly responsible, but the western Pōhue complex was also affected so other hidden structures could exist in the data. Family structures embedded within nesting complexes (or even nesting beaches) that create groups of highly related individuals could be the distortion. More data are required to determine if there could also be assortative mating within nesting complexes, as well as between them.

Relatedness coefficients can also be underestimated when the same samples for which relatedness is being estimated are used to estimate reference allele frequencies [93,94]. The strength of the bias is proportional to the number of samples included, on the order of 1/*N* where *N* is the number of samples. Therefore, when only the adult turtles in this study are used to generate the references, we can expect the strength of the downward bias to be between 0.04 and 0.08. When including hatchlings, which theoretically have the same allele frequencies as their parents, the strength of the bias is between 0.01 and 0.001. Neither scenario fully explains the observed skew, though using hatchlings for the references might exacerbate negative biases due to family structures in the data.

Two more things that could be causing pairwise relatedness coefficients to be downwardly biased are selection and genetic admixture in the population founders [93,95]. Balancing selection and purifying selection acting on different parts of the genome could be creating noise in pairwise relatedness estimates [64], and the experimental loci do not necessarily need to be closely linked to the targets of selection because inbreeding is expected to reduce the rate of homologous recombination, creating large linkage blocks that are passed from generation to generation [4,96]. It is also plausible that hawksbills in the Hawaiian Islands are descended from multiple source populations, and if so then pedigrees would coalesce further back in time, creating a much deeper true reference relative to the present-day sample allele frequencies. How various population-level processes, such as inbreeding, are affecting pairwise relatedness estimates in hawksbill turtles would be clearer with more loci and a better understanding of the genomic architecture (see [97]). Currently, there is no genome sequence for *E. imbricata*, however, a Hawaiian hawksbill turtle was recently selected by the Vertebrate Genomes Project (vertebrategenomesproject.org) to represent this species as the reference genome, and this assembly will enable future research to explore the genomic complexity of this precariously small and non-randomly mating population.

## Data Availability

Data from this study are available through Dryad Digital Repository: https://doi.org/10.5061/dryad.cz8w9gj72 [98]. Custom R code used for data processing can be found on Github (github.com/jh041/loc_gen_acc). The data are provided in electronic supplementary material [99].
